# HIF-1α Inhibition Reduces Nasal Inflammation in a Murine Allergic Rhinitis Model

**DOI:** 10.1371/journal.pone.0048618

**Published:** 2012-11-01

**Authors:** Han Zhou, Xi Chen, Wei-Ming Zhang, Lu-Ping Zhu, Lei Cheng

**Affiliations:** 1 Department of Otorhinolaryngology, The First Affiliated Hospital, Nanjing Medical University, Nanjing, China; 2 Department of Pathology, The First Affiliated Hospital, Nanjing Medical University, Nanjing, China; 3 International Centre for Allergy Research, Nanjing Medical University, Nanjing, China; South Texas Veterans Health Care System and University Health Science Center San Antonio, United States of America

## Abstract

**Background:**

Hypoxia-inducible factor 1α (HIF-1α) is an important regulator of immune and inflammatory responses. We hypothesized that nasal allergic inflammation is attenuated by HIF-1α inhibition and strengthened by HIF-1α stabilization.

**Objective:**

To elucidate the role of HIF-1α in a murine model of allergic rhinitis (AR).

**Methods:**

Mice were pretreated with the HIF-1α inhibitor 2-methoxyestradiol (2ME2) or the HIF-1α inducer cobalt chloride (CoCl_2_) in an established AR murine model using ovalbumin (OVA)-sensitized BALB/c mice. HIF-1α and vascular endothelial growth factor (VEGF) expression in nasal mucosa was measured and multiple parameters of allergic responses were evaluated.

**Results:**

HIF-1α and VEGF levels were locally up-regulated in nasal mucosa during AR. Inflammatory responses to OVA challenge, including nasal symptoms, inflammatory cell infiltration, eosinophil recruitment, up-regulation of T-helper type 2 cytokines in nasal lavage fluid, and serum OVA-specific IgE levels were present in the OVA-challenged mice. 2ME2 effectively inhibited HIF-1α and VEGF expression and attenuated the inflammatory responses. Stabilization of HIF-1α by CoCl_2_ facilitated nasal allergic inflammation. HIF-1α protein levels in nasal airways correlated with the severity of AR in mice.

**Conclusions:**

HIF-1α is intimately involved in the pathogenesis of nasal allergies, and the inhibition of HIF-1α may be useful as a novel therapeutic approach for AR.

## Introduction

Allergic rhinitis (AR) is a common inflammatory disease characterized by nasal itching, sneezing, rhinorrhea, and nasal congestion. It is frequently associated with other inflammatory diseases such as asthma, rhinosinusitis, allergic conjunctivitis, otitis media with effusion, and adenoid hypertrophy [1]. Furthermore, AR is a risk factor for asthma and its prevalence is increasing worldwide [1]. Allergic inflammation in the nasal airways is mediated by T-helper type 2 (Th2) cells together with mast cells, B cells, and eosinophils, as well as a number of inflammatory cytokines and chemokines [2], [3]. Recent evidence shows that hypoxia becomes the normal physiological environment during inflammatory processes [4]. The hypoxia-inducible factor 1 (HIF-1) transcription complex regulates the activation of different immune cells during the inflammatory response [4], [5]. Therefore, the role of HIF-1 in allergic airway inflammation is attracting more attention.

HIF-1 is a heterodimeric transcription complex that regulates cellular responses to low oxygen environments [6]. HIF-1α is the only oxygen-regulated subunit and its stability determines the transcriptional activity of HIF-1. Under normoxic conditions, HIF-1α is rapidly degraded by the ubiquitin-proteasome pathway [7]. In addition to the oxygen-dependent regulation of HIF-1α, several reports have demonstrated that HIF-1α expression is regulated by a variety of cytokines and growth factors via oxygen-independent pathways [8], [9], [10]. Once HIF-1α is activated, it translocates to the nucleus to form a transcriptionally active HIF-1 complex that can stimulate the expression of many target genes such as erythropoietin, some glucose transporters, several glycolytic enzymes, and vascular endothelial growth factor (VEGF) [11]. Functionally, the HIF-1 transcription complex is a major contributor to the inflammatory process [5], [12].

Growing evidence suggests that HIF-1α expression is elevated in the lungs of asthma patients and that it plays an important role in allergic pulmonary inflammatory responses [13], [14]. However, very little is currently known about the exact role of HIF-1α in nasal allergies and inflammation. The present study was designed to examine the role of HIF-1α in nasal mucosa following ovalbumin (OVA) challenge. We hypothesized that acute inhibition of HIF-1α could ameliorate allergic inflammation in the nasal mucosa. On the other hand, up-regulation of HIF-1α by a hypoxia-mimicking agent may deteriorate allergic nasal inflammation. To test our hypothesis, we pretreated mice with the HIF-1α inhibitor 2-methoxyestradiol (2ME2) and the HIF-1α inducer cobalt chloride (CoCl_2_) separately in an established murine model of AR.

## Materials and Methods

### Ethics statement

All experiments involving animals and tissue samples were performed in accordance with the guidelines of the National Institutes of Health (NIH) and Nanjing Medical University with all procedures (2008–0007) approved by the Institutional Animal Care and Use Committee of Nanjing Medical University (Nanjing, China).

### Animals and experimental protocol

Male BALB/c mice, 6 weeks old and free of murine-specific pathogens, were obtained from the Experimental Animal Center of Nanjing Medical University (Nanjing, China). The mice were housed throughout the experiments in a laminar flow cabinet and were maintained on standard laboratory chow ad libitum.

The sensitization and antigen challenges of mice for the murine model of AR were performed as previously described [15]. Briefly, mice were administered 0.5 mg/ml OVA (Grade 5, Sigma-Aldrich, St. Louis, MO, USA) and 20 mg/ml aluminum hydroxide (Sigma-Aldrich) in saline at a dosage of 0.2 ml per mouse by intraperitoneal injection. The sensitization was repeated 3 times at weekly intervals (days 1, 8, and 15) followed by daily intranasal instillations of OVA solution (40 mg/ml in saline) into the nostrils (0.02 ml per mouse) on days 22 to 29 (challenge).

**Figure 1:**
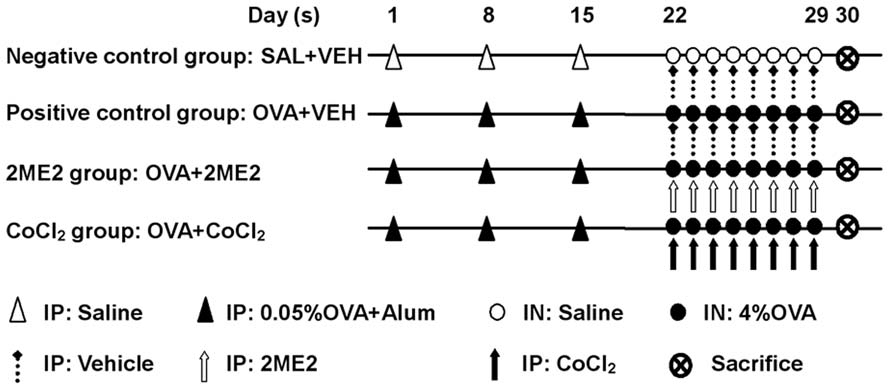
Experimental protocol. BALB/c mice were sensitized with 0.05% OVA and 2% aluminum hydroxide (Alum) solution by intraperitoneal injection on days 1, 8, and 15. Mice were challenged with a 4% OVA solution by daily intranasal instillation from day 22 to day 29. With sensitization and challenge, 2ME2, CoCl_2_ or vehicle (DMSO) solution was given by intraperitoneal injection 2 hours before each intranasal challenge. SAL, saline; VEH, vehicle; IP, intraperitoneal injection; IN, intranasal instillation.

Mice were divided into four groups consisting of 14 mice each, including 1) negative control group: saline-challenged mice with vehicle treatment (SAL+VEH); 2) positive control group: OVA-challenged mice with vehicle treatment (OVA+VEH); 3) 2ME2 group: OVA-challenged mice with 2ME2 treatment (OVA+2ME2); 4) CoCl_2_ group: OVA-challenged mice with CoCl_2_ treatment (OVA+CoCl_2_). The procedures for allergen sensitization and treatment are summarized in **Figure 1**. Along with sensitization and challenge, 2ME2 (30 mg/kg body weight/day, Sigma-Aldrich), CoCl_2_ (9.8 mg/kg body weight/day, Sigma-Aldrich), or vehicle (dimethyl sulfoxide, DMSO) in 0.2 ml saline was given by intraperitoneal injection 2 hours before each intranasal challenge.

A concentrated stock solution of 2ME2 was prepared in DMSO and then diluted in saline to prepare a solution of 2ME2 that was administrated intraperitoneally to the 2ME2 group. For the other three groups, the solution of CoCl_2_ or vehicle only contained concentrations of DMSO equivalent to that used for 2ME2 treatment.

### Evaluation of nasal symptoms and sample preparation

On day 29 after the final intranasal administration of OVA or physiological saline, mice were placed individually in observation cages. The number of sneezes and the frequency of nasal rubbing (nasal scratching movements) were counted for 10 minutes by blinded observers. Twenty-four hours after the final intranasal challenge, mice were deeply anesthetized by an intraperitoneal injection of sodium pentobarbital. Their abdomen was opened and blood samples were obtained via the inferior vena cava. Mice were then killed by exsanguination.

After the mice were killed, nasal lavages were performed following partial tracheal resection using 22-gauge catheters. A catheter was inserted into the nasopharynx from the tracheal opening and the nasal passages were gently perfused with 1 ml of phosphate-buffered saline (PBS) from the choana to the nostril. Nasal lavage fluid (NLF) was collected from the nares. NLF was centrifuged for 10 minutes at 3500 rpm at 4°C, and the supernatants were collected. Blood samples were left for 30 minutes at room temperature, and serum was obtained after centrifugation. Samples were stored at −80°C.

The nasal mucosa from 10 mice from each group was obtained using a small curette, immediately immersed into liquid nitrogen, and stored at −80°C. Six specimens from each group were dissected for protein isolation, and mRNA was extracted from the remaining samples.

### Histological analysis and immunohistochemistry

The decapitated heads of four mice from each group were fixed in 10% neutral buffered formalin for 24 hours at 20°C. Specimens were decalcified for 21 days in 10% EDTA-Na and embedded in paraffin. Paraffin-embedded nasal cavities were coronally sectioned (4 µm thick) approximately 5 mm from the nasal vestibule. Each section was deparaffinized and stained with hematoxylin and eosin (H&E) to evaluate general morphology. The same region of the coronal slice of the septal mucosa in each mouse was observed blindly by light microscopy. Five high-power fields (magnification, 400×) were randomly selected from each section and infiltrating eosinophils were counted. The number of infiltrating eosinophils in each animal was expressed as the mean value from the five fields.

Immunostaining for HIF-1α and VEGF was performed using *Ultrasensitive*™ immunohistochemistry S-P kits (Maixin Biology Corporation, Fuzhou, China). Sections (4 µm thick) were dewaxed, washed in Tris buffered saline (TBS), and incubated with 3% H_2_O_2_ in methanol for 20 minutes to block endogenous peroxidases. Sections were then washed with TBS and incubated with 5% skim milk in TBS for 20 minutes. Blocked sections were incubated with antibodies against HIF-1α (monoclonal mouse anti-HIF-1α antibody; Novus Biologicals, Littleton, CO, USA, NB100-105) or VEGF (rabbit polyclonal anti-VEGF antibody; Santa Cruz Biotechnology, Santa Cruz, CA, USA, sc-152) for 90 minutes at 37°C. Secondary antibody was applied to each section for 10 minutes at room temperature, and the slides were rinsed in PBS (pH 7.4) three times after every incubation step. The slides were counterstained with hematoxylin, mounted, and observed under light microscopy.

### Measurement of cytokines in NLF and OVA-specific IgE in sera

Levels of the Th2 cytokines IL-4 and IL-5 were quantified in NLF supernatants using enzyme-linked immunosorbent assays (ELISAs) according to the manufacturer's protocol (ELISA kit from R&D Systems Inc., Minneapolis, MN, USA). After measuring the optical density at 450 nm, the concentrations of IL-4 and IL-5 were determined by interpolation from a standard curve. Using a similar method, serum anti-OVA IgE concentrations were measured using an anti-OVA IgE-specific ELISA kit (Shibayagi, Gunma, Japan).

### Western blot analysis of HIF-1α and VEGF in nasal mucosa

Western blot analyses were performed as previously described with some modifications [16]. Protein extractions from whole-cell lysates were obtained using a total protein extraction kit (KeyGEN Biotech., Nanjing, China) and further centrifuged at 14,000 rpm at 4°C for 15 minutes. Supernatants were used as whole cell protein extracts and protein concentrations were determined using bicinchoninic acid (BCA) protein assay kits (Beyotime Institute of Biotechnology, Jiangsu, China). Equal amounts of protein (60 µg) were loaded on SDS-PAGE gels. Following electrophoresis, proteins were transferred to polyvinylidene difluoride (PVDF) membranes. Membranes were blocked and incubated with a primary antibody overnight at 4°C. The primary antibodies used were monoclonal mouse anti-HIF-1α (Novus Biologicals, NB100-105, 1∶250), rabbit polyclonal anti-VEGF (Santa Cruz Biotechnology, sc-507, 1∶100), and monoclonal mouse anti-β-actin (Boster, Wuhan, China, BM0627, 1∶400). PVDF membranes were incubated with secondary antibodies (Cell Signaling Technology, Beverly, MA, USA) for 1 hour at room temperature. Immunoblots were probed using ECL Plus chemiluminescence kits (Thermo Fisher Scientific Inc., Rockford, IL, USA) and visualized using a Molecular Imager Gel Doc™ XR+ imaging system (Bio-Rad Laboratories, Hercules, CA, USA). Data were analyzed using Quantity one 4.6.2 (Bio-Rad) software.

### Real-time RT-PCR for HIF-1α and VEGF in nasal mucosa

Total RNA was extracted using TRIzol reagent (Invitrogen, Carlsbad, CA, USA) following the manufacturer's instructions. For quantitative RT-PCR, reverse transcription was performed using 2 µg of total RNA, an oligo (dT) 18 primer, and M-MLV reverse transcriptase (Takara, Syuzou, Shiga, Japan). mRNA levels were determined using a Mastercycler® RealPlex2 real-time PCR System (Eppendorf, Hamburg, Germany) and SYBR® *Premix EX Taq*™ (Takara). The sequences of primers used were: HIF-1α forward: 5′-TGCTCATCAGTTGCCACTT-3′, reverse: 5′-TGGGCCATTTCTGTGTGTA-3′; VEGF forward: 5′-CCAAGTGGTCCCAGGCTGCACC-3′, reverse: 5′- GGTTAATCGGTCTTTCCGGTGAG-3′; and GAPDH forward: 5′- ACCACAGTCCATGCCATCAC-3′, reverse: 5′- TCCACCACCCTGTTGCTGTA-3′. The PCR mixtures contained 2× SYBR Green Master Mix (Takara), 10 µM primers, and 50 ng cDNA in a 20-µl volume. Reactions were heated to 95°C for 30 seconds followed by 40 cycles of 95°C for 3 seconds and 60°C for 30 seconds. All PCR reactions were performed in triplicate. PCR product specificity was evaluated by melting curve analysis and by separation in agarose gels. Using five dilutions of cDNA, the linearity of PCR amplification was controlled. HIF-1α or VEGF levels were expressed as fold increases or decreases relative to GAPDH expression. The mean values of the replicates for each sample were calculated and expressed as cycle threshold (Ct). Gene expression levels were calculated as the difference (ΔCt) between the Ct value of HIF-1α or VEGF and the Ct value of GAPDH. Fold changes in HIF-1α or VEGF mRNA were determined as 2^−ΔΔCt^.

### Statistical analysis

Data were analyzed using GraphPad Prism version 5.0 (GraphPad Inc., San Diego, CA, USA) and presented as means±SEM. One-way ANOVA followed by Dunnett's tests were used to determine significant differences between treatment groups. Significance levels were set at p<0.05.

## Results

### Effects of 2ME2 and CoCl2 on nasal symptoms in a murine AR model

**Figure 2:**
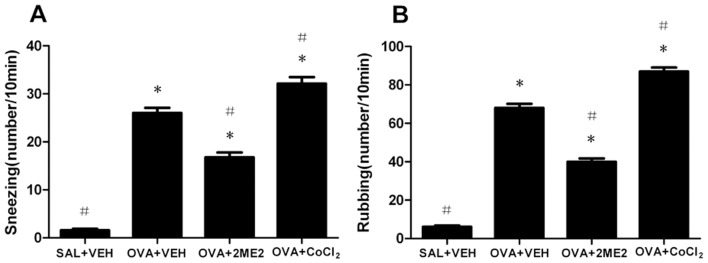
Effects of 2ME2 and CoCl_2_ on nasal symptoms. The number of sneezes (A) and nasal rubbing frequencies (B) were observed over 10 minutes immediately following the final challenge. Values represent means±SEM of 14 mice in each group. *Significantly different from negative control, p<0.05; ^#^significantly different from positive control, p<0.05.

The number of sneezes and the frequency of nasal rubbing in the positive control group were 26.0±1.10 and 68.0±2.11, respectively, and were significantly elevated compared to those for the negative control group (both p<0.05). The incidence of sneezing after the final intranasal OVA challenge was reduced by treatment with 2ME2 (16.8±0.98, p<0.05) and increased by treatment with CoCl_2_ (32.1±1.33, p<0.05) compared to the positive control group. Moreover, the change in nasal rubbing correlated with that of sneezing (39.9±1.81 and 86.9±2.14, respectively, both p<0.05). **Figure 2** illustrates these changes in OVA-induced nasal symptoms.

### Histology of nasal mucosa and eosinophil infiltration

**Figure 3:**
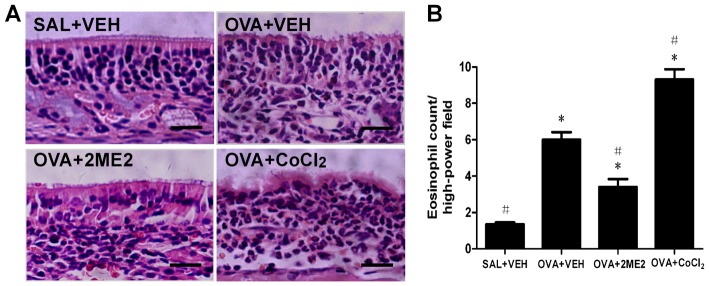
Histology of nasal mucosa and eosinophil infiltration. (A) Histological findings of the nasal mucosa in each group (magnification 400×, scale bar = 20 µm). (B) Eosinophil counts in the nasal mucosa were significantly reduced by treatment with 2ME2 and increased by treatment with CoCl_2_ compared to the positive control group. *Significantly different from negative control, p<0.05; ^#^significantly different from positive control, p<0.05.

Histological analysis of the nasal mucosa from OVA-challenged mice revealed that numerous inflammatory cells, including eosinophils, infiltrated the nasal mucosa. Furthermore, the nasal tissues were thickened compared to the negative control (**Figure 3A**). The number of eosinophils in the positive control group increased compared to the negative control group (p<0.05, **Figure 3B**). Allergic mice treated with 2ME2 showed marked reductions in the number of infiltrating eosinophils, while CoCl_2_ treatment enhanced eosinophil infiltration into the nasal mucosa (both p<0.05, **Figure 3B**). These findings were consistent with the changes in the nasal symptoms following 2ME2 or CoCl_2_ treatments.

### Effects of 2ME2 and CoCl2 on cytokine levels in NLF and OVA-specific IgE in sera

**Figure 4:**
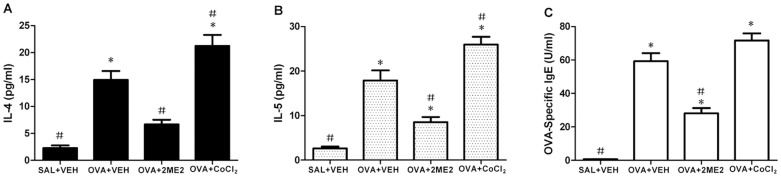
Effects of 2ME2 and CoCl_2_ on expression of IL-4 and IL-5 in NLF and OVA-specific IgE in sera. Sampling was performed 24 h after the last challenge in saline/OVA-treated mice administered with either vehicle (DMSO), 2ME2, or CoCl_2_. Levels of IL-4 (A) and IL-5 (B) in NLF and OVA-Specific IgE (C) in sera were analyzed using ELISAs (n = 10 mice). Values represent means±SEM. *Significantly different from negative control, p<0.05; ^#^significantly different from positive control, p<0.05.

To determine the effects of 2ME2 and CoCl_2_ on Th2 inflammation in OVA-challenged mice, we measured levels of the Th2 cytokines IL-4 and IL-5 in NLF. As shown in **Figure 4A and 4B**, IL-4 and IL-5 levels in NLF were significantly elevated following the last OVA challenge compared to those in the negative control mice. The increased IL-4 and IL-5 levels after the OVA challenge were reduced significantly by administration of 2ME2. Administration of CoCl_2_, on the contrary, resulted in increased Th2 cytokine levels.

Serum levels of OVA-specific IgE in the OVA-sensitized group were significantly higher than in the unsensitized group. Administration of 2ME2 significantly decreased the serum levels of OVA-specific IgE (**Figure 4C**, p<0.05). CoCl_2_ treatment increased the serum levels of OVA-specific serum IgE. However, the levels did not reach statistically significant values (**Figure 4C**, p>0.05).

### Effects of 2ME2 and CoCl2 on HIF-1α and VEGF expression in OVA-challenged mice

**Figure 5:**
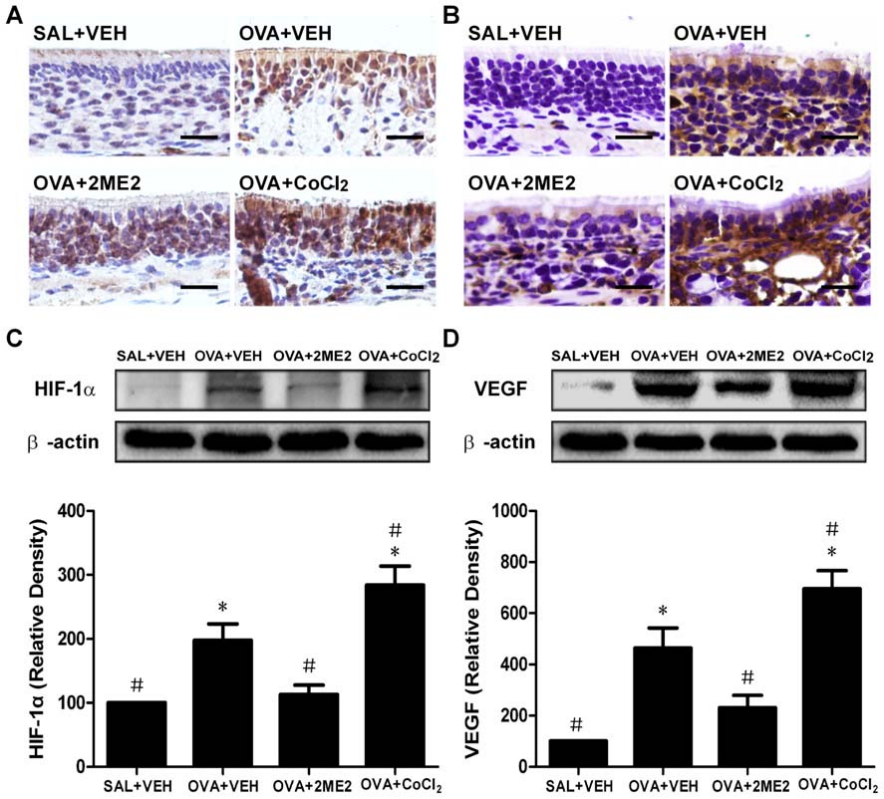
Immunohistochemical and Western blot analysis of HIF-1α and VEGF expression in OVA-challenged mice. (A and B) Immunostaining for HIF-1α and VEGF in nasal mucosa following the last challenge. Positive HIF-1α signals are brown and are predominantly nuclear and VEGF is predominantly cytoplasmic (magnification 400×, scale bar = 20 µm). (C and D) Representative Western blot analysis showing HIF-1α and VEGF (with β-actin as a loading control) expression in the nasal mucosa 24 h after the last challenge. All densitometric analyses are presented as the relative ratio of each molecule to β-actin and the ratio in negative control mice was set to 100. Values represent means±SEM (n = 6 mice). *Significantly different from negative control, p<0.05; ^#^significantly different from positive control, p<0.05.

Immunohistochemical analysis of nasal cavity sections revealed that both HIF-1α and VEGF were extensively up-regulated in the nasal mucosa following allergen challenge. The positive protein signals were seen as brown granular deposits within nasal mucosal epithelium cells. HIF-1α was predominantly nuclear, although some cytoplasmic staining was observed. VEGF was expressed predominantly in the cytoplasm or in cell membranes. After 2ME2 treatment, there were fewer cells with condensed staining of HIF-1α and VEGF. Furthermore, the immunostaining of both HIF-1α and VEGF were more intense in the nasal cavity of the CoCl_2_ group than in the positive control group (**Figure 5A and 5B**).

The immunohistochemical results were confirmed by Western blotting. Western blot analysis showed an up-regulation of HIF-1α 24 h after the last OVA challenge, and it was significantly inhibited by 2ME2 (**Figure 5C**, both p<0.05). VEGF was up-regulated at 24 h after the last OVA challenge and significantly reduced in the 2ME2 group (**Figure 5D**, both p<0.05). On the other hand, in the CoCl_2_ group, there were significantly higher HIF-1α levels than in the positive control group (**Figure 5C**, p<0.05). Moreover, CoCl_2_ treatment also elevated VEGF protein levels compared to the positive control group (**Figure 5D**, p<0.05).

**Figure 6:**
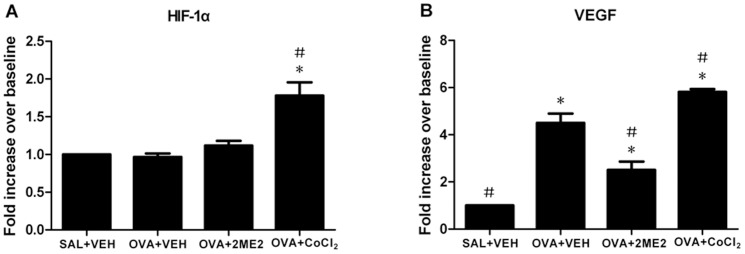
Real-time RT-PCR analysis of HIF-1α and VEGF mRNA expression in nasal mucosa. Real-time RT-PCR was performed to detect HIF-1α (A) and VEGF (B) mRNA in nasal mucosa. Relative target gene expression was normalized to GADPH, an internal control. Values represent the ratios of various treatments to the negative control group. Values represent means±SEM of four mice in each group. *Significantly different from negative control, p<0.05; ^#^significantly different from positive control, p<0.05.

As shown in **Figure 6**, HIF-1α mRNA levels from the nasal mucosa had no significant differences between the positive control and 2ME2 groups. However, HIF-1α mRNA levels were up-regulated after CoCl_2_ treatment compared to the positive control mice. OVA challenge markedly up-regulated nasal mucosa VEGF mRNA expression, while 2ME2 treatment suppressed its expression compared with the saline-challenged mice. However, pretreatment with CoCl_2_ induced a strong up-regulation of VEGF mRNA levels.

## Discussion

Emerging evidence has demonstrated that HIF-1α contributes to allergic immune responses and bronchial asthma [5], [13], [14], [17]. Our studies using a murine model of AR confirm and extend previous observations suggesting that HIF-1α plays an important role in regulating nasal allergic immune responses. First, we showed that HIF-1α and VEGF levels are up-regulated in nasal mucosa during AR in mice. The inflammatory responses to OVA challenge, including nasal symptoms, inflammatory cell infiltration, eosinophil recruitment, and up-regulation of IL-4 and IL-5 in NLF and OVA-specific IgE levels in sera, were observed in the OVA-challenged group. Second, the inflammatory responses to OVA challenge were attenuated by treatment with 2ME2, a HIF-1α inhibitor, and strengthened by treatment with CoCl_2_, a HIF-1α inducer. Therefore, we demonstrated that HIF-1α protein levels in nasal airways correlate with the severity of AR in mice. Taken together, these observations suggest that HIF-1α is intimately involved in the pathogenesis of AR.

HIF-1α activation is required for inflammatory reactions associated with infections and allergies [5]. In inflammatory tissues, HIF-1α accumulates in response to and is activated by various mediators, including hypoxia, nitric oxide, inflammatory cytokines, growth factors, reactive oxygen species, apoptotic cell debris, infectious pathogens, and allergens [4], [5], [8], [9], [10], [13]. Although regulated by differential intracellular signalling mechanisms, HIF-1α protein accumulates in both basophils and mast cells induced by pro-allergic (IgE-mediated) stimulation, and both the cells are crucial effectors in Th2 cell-dependent IgE-associated allergic disorders [18], [19], [20], [21]. Moreover, the roles of this factor in inflammatory reactions associated with allergies have recently become evident. In addition to the promotion of angiogenesis, the functional roles of HIF-1α also include supporting energy metabolism in inflammatory cells and promoting pro-inflammatory cytokine expression and immune cell adhesion [4], [5], [10], [22], [23]. These functions are all critical for immune and inflammatory responses.

VEGF, one of the most important target genes preferentially regulated by HIF-1α, is also an important multifunctional angiogenic regulator capable of promoting proliferation and migration of endothelial cells and increasing vascular permeability [24]. VEGF is also a mediator of vascular and extravascular remodeling and inflammation and plays a crucial role in adaptive Th2 inflammation [25]. Several studies have shown that increased HIF-1α expression causes overproduction of VEGF, which results in the leakage of plasma proteins, inflammatory mediators, and inflammatory cells into the extravascular space of the airway [13], [19]. In keeping with these observations, our study demonstrated that changes in VEGF mRNA and protein expression correlated with HIF-1α protein levels, suggesting VEGF expression might be regulated by HIF-1α in the nasal mucosa during allergic inflammation. Therefore, one likely mechanism for the roles of HIF-1α in the pathogenesis of AR is the enhancement of Th2-mediated sensitization and inflammation via up-regulation of nasal VEGF expression.

2ME2, an endogenous metabolite of estradiol, inhibits HIF-1α and is an anti-angiogenic and antitumor agent. Although its exact mechanism of action is not yet known, the HIF-1α inhibitory properties of 2ME2 are thought to be related to microtubule depolymerization [26], [27]. It is unclear if the 2ME2 effects are entirely mediated via HIF-1α inhibition. However, recently published reports have provided *in vitro* and *in vivo* evidence that 2ME2 has a direct effect on HIF-1α inhibition and is not the result of a “side effect” of mitotic arrest [26], [27]. 2ME2 in moderate concentrations has no direct toxic effects on slowly dividing non-tumor cells, but it does lead to reliable inhibition of HIF-1α [13], [27], [28]. Furthermore, 2ME2 treatment can reduce the levels of nuclear and total HIF-1α protein in a dose-dependent manner [26]. 2ME2 can reduce the levels of HIF-1α in the lung tissues of OVA-challenged mice through the inhibition of HIF-1α translation and its nuclear translocation, thereby suppressing VEGF expression. Such HIF-1α inhibition can ameliorate allergic airway disease [13].

Consistent with previous observations, HIF-1α protein levels in the nasal mucosa were substantially elevated in our mouse model of OVA-induced AR, suggesting that HIF-1α was activated. The increased HIF-1α levels were significantly reduced following administration of 2ME2. In addition, the increased VEGF levels in nasal mucosa following OVA challenge were also significantly reduced by administration of 2ME2 *in vivo*. Meanwhile, the titer of OVA-specific IgE was significantly lower following 2ME2 treatment. This decrease in IgE may have been caused by inhibitory effects on B-cell activation and reduced release of Th2 cytokines, which correlated well with the changes in IL-4 and IL-5 levels in NLF. IL-4 promotes Th2 cell differentiation and induces B cells to switch to IgE production [29]. IL-5 increases eosinophilic inflammation and airway infiltration [30]. As major Th2 cytokines, both of them are key mediators in allergic inflammation. In addition, eosinophils play a central role in the pathogenesis of allergic inflammation [31]. Our present findings show that 2ME2 prevented infiltration of OVA-induced inflammatory cells into the upper airways as shown by a significant drop in eosinophil counts in the nasal mucosa. Therefore, because of the reduction in nasal mucosa eosinophilia, IL-4 and IL-5 levels in NLF, and OVA-specific IgE levels in sera, 2ME2 treatment likely inhibits the nasal allergic responses. Because there was also a significant decrease in sneezing and nasal rubbing counts, this inhibition of nasal allergies may also contribute to the clinical alleviation of AR. Taken together, these findings suggest that HIF-1α inhibition by 2ME2 suppresses VEGF expression and greatly reduces nasal allergic inflammation.

To further ascertain whether HIF-1α play a critical role in AR, we used CoCl_2_ [32]. CoCl_2_ is widely used to mimic hypoxic conditions in tissues and can activate the PI3K pathway, which acts upstream of a translation stimulation signal to enhance the translation of HIF-1α mRNA [32], [33]. Meanwhile, CoCl_2_ can also prevent the rapid degradation of HIF-1α by inhibiting HIF-1α prolyl hydroxylases [34]. In our study, CoCl_2_ increased HIF-1α and VEGF levels as compared with vehicle-treated mice. Moreover, the CoCl_2_-treated animals also showed qualitatively more eosinophil infiltration and significantly higher levels of IL-4 and IL-5 in NLF than in the positive control group. Serum OVA-specific IgE levels were higher following CoCl_2_ treatment, but the difference was not significant, which may due to the relatively small sample size of our study. Hence, the deterioration of nasal symptoms in the CoCl_2_ group may be associated with enhanced nasal allergic inflammation via excitation of HIF–mediated pathways. From these results, we further confirmed that up-regulation of HIF-1α contributes to nasal allergic inflammation.

In the present study, HIF-1α mRNA was induced in the nasal mucosa of OVA-challenged mice, but there were no significant differences among the negative control, the positive control, and 2ME2 groups. Meanwhile, CoCl_2_ treatment increased HIF-1α mRNA levels 1.8 fold compared to the negative control. These findings did not correspond to the protein expression levels in our study. Two reasons may account for these inconsistent results. First, inflammatory stress caused by allergic inflammation results in the stabilization and accumulation of HIF-1α protein [5], [18]. Thus, HIF-1α protein levels may increase upon allergic stimulation without an accompanying increase in HIF-1α mRNA transcription. Furthermore, 2ME2 down-regulates HIF-1α at the posttranscriptional level [26], which does not affect the expression of HIF-1α mRNA. CoCl_2_ may stabilize HIF-1α protein. Therefore, HIF-1α protein expression was much higher than was the mRNA level. Second, *in vivo* studies showed that robust expression of HIF-1α mRNA in response to hypoxia is transient in the tissues [35]. Thus, we likely did not detect increased HIF-1α mRNA expression. Moreover, continuous high levels of HIF-1α may regulate its mRNA levels through a negative feedback loop. Therefore, this may also account for the relatively low expression of HIF-1α mRNA in our study.

HIF-1α and VEGF were locally up-regulated in a murine AR model in a manner similar to that observed in lungs following allergic asthma. The mechanism of HIF–mediated pathways *in vivo* has not been confirmed completely. However, the current study suggests that nasal HIF-1α variability correlated with AR conditions. In addition, 2ME2 effectively reduced OVA-induced nasal symptoms, eosinophil infiltration, IL-4 and IL-5 levels, and OVA-specific IgE production potentially via inhibition of the HIF–VEGF signaling pathway. Stabilization of HIF-1α by CoCl_2_, however, could facilitate these nasal inflammatory responses. HIF-1α is intimately involved in the pathogenesis of nasal allergies and inhibition of HIF-1α may be useful as a novel therapeutic approach in AR.
